# Brivanib Attenuates Hepatic Fibrosis *In Vivo* and Stellate Cell Activation *In Vitro* by Inhibition of FGF, VEGF and PDGF Signaling

**DOI:** 10.1371/journal.pone.0092273

**Published:** 2014-04-07

**Authors:** Ikuo Nakamura, Kais Zakharia, Bubu A. Banini, Dalia S. Mikhail, Tae Hyo Kim, Ju Dong Yang, Catherine D. Moser, Hassan M. Shaleh, Sarah R. Thornburgh, Ian Walters, Lewis R. Roberts

**Affiliations:** 1 Division of Gastroenterology and Hepatology, Mayo Clinic College of Medicine, Rochester, Minnesota, United States of America; 2 Bristol-Myers Squibb, Wallingford, Connecticut, United States of America; University of Navarra School of Medicine and Center for Applied Medical Research (CIMA), Spain

## Abstract

**Background and Aims:**

Brivanib is a selective inhibitor of vascular endothelial growth factor receptor (VEGFR) and fibroblast growth factor receptor (FGFR) tyrosine kinases, which are both involved in mechanisms of liver fibrosis. We hypothesized that inhibition of VEGFR and FGFR by brivanib would inhibit liver fibrosis. We therefore examined the effect of brivanib on liver fibrosis in three mouse models of fibrosis.

**Methods:**

*In vivo*, we induced liver fibrosis by bile duct ligation (BDL), chronic carbon tetrachloride (CCl_4_), and chronic thioacetamide (TAA) administration. Liver fibrosis was examined by immunohistochemistry and Western immunoblotting. *In vitro*, we used LX-2 human hepatic stellate cells (HSCs) to assess the effect of brivanib on stellate cell proliferation and activation.

**Results:**

After *in vivo* induction with BDL, CCl_4_, and TAA, mice treated with brivanib showed reduced liver fibrosis and decreased expression of collagen Iα1 and α-smooth muscle actin in the liver. *In vitro*, brivanib decreased proliferation of HSCs induced by platelet-derived growth factor (PDGF), VEGF, and FGF. Brivanib also decreased stellate cell viability and inhibited PDGFBB-induced phosphorylation of its cognate receptor.

**Conclusion:**

Brivanib reduces liver fibrosis in three different animal models and decreases human hepatic stellate cell activation. Brivanib may represent a novel therapeutic approach to treatment of liver fibrosis and prevention of liver cancer.

## Introduction

Hepatic stellate cells (HSCs) are activated in response to liver damage. With repeated liver injury, HSCs trans-differentiate into myofibroblast-like cells that contribute to the development of hepatic fibrosis by production of collagen [1], [2]. Activated HSCs and myofibroblasts also produce a number of profibrotic cytokines and growth factors that perpetuate the fibrotic process through paracrine and autocrine effects [3]. In the context of ongoing liver injury, liver fibrosis progresses to cirrhosis and is associated with neoplastic transformation to hepatocellular carcinoma (HCC) [4]. Growth factors elevated in liver fibrosis and cirrhosis that have been implicated in carcinogenesis include transforming growth factor alpha (TGF-α), epidermal growth factor (EGF), insulin-like growth factor 1 (IGF1), vascular endothelial growth factor (VEGF), fibroblast growth factor (FGF) and platelet-derived growth factor (PDGF) [5], [6], [7], [8]. PDGF-BB and transforming growth factor beta 1 (TGF-β1) have been shown to play key roles in fibrogenesis [9], [10]. The genetic over-expression of PDGF leads to liver fibrosis in mice [3], [11]. TGF-β promotes HSC transformation into myofibroblasts, stimulates the synthesis of extracellular matrix proteins and inhibits their degradation [12]. In addition, there is evidence that FGF and VEGF signaling both play roles in liver fibrogenesis. Liver fibrosis resulting from chronic carbon tetrachloride (CCl_4_) exposure has been shown to be markedly decreased in FGF1/FGF2-deficient mice [13]. Furthermore, PTK/ZK, a pan-VEGFR tyrosine kinase inhibitor, was shown to attenuate collagen deposition and α-smooth muscle actin (α-SMA) expression in CCl_4_-induced mouse liver fibrosis in both a ‘prevention’ and ‘treatment’ dosing scheme. VEGF and FGF are considered the most important of the angiogenic factors involved in vascularization of hepatocellular carcinoma (HCC) and are attractive targets for therapy of HCC [14], [15]. Brivanib alaninate is a potent and selective inhibitor of both the VEGFR and FGFR tyrosine kinases which has been evaluated in clinical trials for treatment of HCC [14], [16], [17], [18], [19]. Given the evidence for roles for both FGF and VEGF signaling in liver fibrogenesis, we hypothesized that brivanib would significantly inhibit liver fibrogenesis. Since the effect of brivanib on liver fibrosis has not been studied, the aim of this study was to explore the effect of brivanib on liver fibrosis in three different mouse models, as well as the effects of brivanib on TGF-β, PDGF, VEGF, and FGF-induced proliferation and activation of hepatic stellate cells.

## Materials and Methods

### Ethics Statement

The care and use of animals for this study complied fully with relevant governmental and institutional requirements, guidelines and policies. Mice were obtained from The Jackson Laboratory (Maine, USA). They were maintained in a temperature-controlled (22°C), pathogen-free environment and fed a standard rodent chow diet and water ad libitum. The study was reviewed and approved by the Institutional Animal Care and Use Committee (IACUC) at the Mayo Clinic.

### Chemicals and antibodies

Rabbit anti-α-smooth muscle actin (α-SMA) (1/200 dilution, Novus, Littleton, CO, USA) was used for immunohistochemistry. The following antibodies were used for Western immunoblotting: β-actin and α-SMA (A5316 and A5228, respectively, Sigma-Aldrich, St. Louis, MO, USA), total PDGFRβ and phosphorylated PDGFRβ (#3169 and #3161, respectively, Cell Signaling Technology Inc, Danvers, MA). Protease inhibitor cocktail set III (539134, Calbiochem, San Diego, CA, USA), PVDF membrane and 4–15% Tris HCl gel (BioRad, Richmond, CA, USA), and ECL-enhanced chemiluminescence reagents (Denville Scientific Inc., Metuchen, NJ, USA) were used. To study the effect of brivanib on growth factor signaling pathways, human recombinant PDGF-BB (P3201, Sigma-Aldrich, St. Louis, MO, USA), VEGF, FGF and TGF-β1 (293-VE, 233-FB and 5036-WN, respectively, R&D Systems, Minneapolis, MN, USA) were used. For real time PCR, the RNAeasy Mini Kit (Qiagen, Valencia, CA, USA) and High Capacitv cDNA Reverse Transcription kit (Applied Biosystems, Foster City, CA, USA) were used. The following primers for real time PCR were obtained from Applied Biosystems (Foster City, CA, USA): *collagen Iα1* (catalog number Mm00801673); *PDGFB* (Mm440677_m1); *PDGFRB* (Mm00435546_m1); *TGFB1* (Mm1178820_m1); *TGFBR2* (Mm00436977_m1); *FGF2* (Mm00433287_m1); *FGFR2* (Mm01269930_m1); *VEGFA* (Mm01281449_m1); *VEGFR2* (Mm01222921_m1) and *18s* rRNA (4352930E).

### Culture and drug treatment of LX-2 hepatic stellate cells

The LX-2 primary human hepatic stellate cell (HSC) line (provided by Dr. Vijay H. Shah, Mayo Clinic, Rochester, MN with kind permission from Dr. Scott L. Friedman, Mount Sinai School of Medicine, NY) was cultured in serum supplemented DMEM [20]. To study the effect of brivanib on TGF-β signaling in HSCs, the cells were incubated in medium with 1% FBS for 24 hours and stimulated with 2 ng/ml TGF-β1 for 24 hours.

### Animals

C57BL/6 mice were purchased from The Jackson Laboratory (Maine, USA). Mice were maintained in a temperature-controlled (22°C), pathogen-free environment and fed a standard rodent chow diet and water ad libitum. The care and use of the animals for these studies were reviewed and approved by the Institutional Animal Care and Use Committee at the Mayo Clinic.

### Animal models of liver fibrosis

Liver fibrosis was induced by 1) bile duct ligation, 2) chronic carbon tetrachloride administration, or 3) thioacetamide administration. Following each treatment, animals were sacrificed to assess measures of liver fibrosis.

#### 1) Bile duct ligation (BDL)

Cohorts of male mice 6–8 weeks old were subjected to BDL performed as described previously [21]. Beginning 7 days after BDL, placebo or brivanib (25 or 50 mg/kg) was administered daily by the oral route for 7 days. Animals were sacrificed 14 days after BDL.

#### 2) Chronic carbon tetrachloride (CCl_4_) administration

Male mice 4–6 weeks old were treated twice a week for 4 weeks with a total of 8 intraperitoneal (i.p.) injections of 0.5 ml/kg CCl_4_ (diluted 1∶10) to induce liver fibrosis. From the onset of CCl_4_ injections, placebo or brivanib (25, 50, or 100 mg/kg) was administered orally on 5 consecutive days with two days off on weekends. Animals were sacrificed 4 weeks after initiation of the experiment.

#### 3) Thioacetamide (TAA) administration

Male mice 4–6 weeks of age were treated 3 times a week with a total of 12 intraperitoneal (i.p.) injections of 150 ml/kg TAA. At the onset of TAA treatment, placebo or brivanib (25 or 50 mg/kg) was administered orally on 5 consecutive days with weekend breaks. The animals were sacrificed 4 weeks after the start of the injections.

### Assessment of liver injury, induced fibrosis and response to brivanib

To measure serum alanine transaminase (ALT), mice were anesthetized and blood was collected at the time of sacrifice. Liver tissue was frozen in liquid nitrogen for later analysis. Part of each liver sample was also fixed in 10% formalin, embedded in paraffin, and stained with hematoxylin-eosin (H&E) for histological analysis.

### Histology for Sirius red and Masson's trichrome staining

To assess the extent of induced fibrosis and response to brivanib or placebo treatment, liver samples were stained with Sirius red and Masson's trichrome using standard techniques. The number of bands of bridging fibrosis per high power field was quantified.

### RNA isolation and real-time RT-PCR analysis

Total RNA was prepared from liver tissue samples using the RNAeasy Mini Kit. High Capacity cDNA Reverse Transcription kit was used for the production of complementary DNA. For quantitative real-time PCR analysis, primers for *collagen Iα1*, *PDGFB*, *PDGFRB*, *TGFB1*, *TGFBR2*, *FGF2*, *FGFR2*, *VEGFA*, and *VEGFR2* were used in an ABI 7900 system with the following profile: 95°C for 10 minutes followed by 40 cycles of 15 seconds at 95°C and 60 seconds at 60°C. Each mRNA level was normalized by comparison to 18S ribosomal RNA level in the same sample.

### Western immunoblotting

#### Effect of TGF-β1

LX-2 HSCs were plated on 6-well plates at 1×10^5^ cells per well and incubated overnight. The cells were then serum-starved for 24 hours in 1% FBS. After that, TGF-β1 was added and the cells were incubated for 24 hours, followed by preparation of protein lysates in lysis buffer (FNN0011, Invitrogen, CA, USA). Protease inhibitor cocktail set III and PMSF were added to the lysis buffer immediately before use. To determine the effect of brivanib on the activation of α-SMA by TGF-β1, LX-2 cells were incubated with increasing concentrations of brivanib for 1 hour before stimulation with 2 ng/ml TGF-β1. Equal amounts of protein (20 µg/lane) were separated by electrophoresis on a 4–15% Tris HCl gel and transferred to PVDF membrane. Blots were probed with monoclonal antibodies against α-SMA and β-actin. To detect antigen-antibody complexes, the membrane was incubated in horseradish peroxidase-conjugated secondary antibodies at 4°C overnight. Immune complexes were visualized using ECL-enhanced chemiluminescence reagents and film.

#### Effect of PDGF-BB

LX-2 HSCs were plated in 10 cm dishes at 5×10^5^ cells per dish in 10% FBS-supplemented DMEM and incubated for 24 hours. Cells were starved in serum-free medium for 24 hours, followed by incubation with different doses of PDGF-BB (0, 5, 10 ng/ml) for 1 or 5 minutes. Protein lysates were prepared using lysis buffer. For Western immunoblotting, we used monoclonal antibodies against phosphorylated PDGFRβ, total PDGFRβ and β-actin. To determine the effect of brivanib on PDGF-BB induced phosphorylation of PDGFRβ, HSCs were serum-starved and treated with varying doses of brivanib. Two hours later, cells were incubated for 5 minutes with the optimal dose of 5 ng/ml PDGF-BB. Protein lysates were prepared for immunoblotting.

### Cell proliferation assay

Cell proliferation was measured in HSCs using the BrdU incorporation assay (Roche, Indianapolis, IN) according to the manufacturer's instructions. LX-2 HSCs were plated into 96-well plates at 1,000 cells per well and incubated in 10% fetal bovine serum (FBS) overnight. The next day, the cells were starved for 24 hours and each growth factor: PDGF, TGF-β1, FGF, or VEGF was added at different concentrations. The cells were incubated for 72 hours and BrdU incorporation was then measured. Each experiment was performed in six replicates at least three times.

### Cell viability

Viability was measured in LX-2 cells using the Cell Counting Kit-8 (CCK-8) from Dojindo Molecular Technology, Inc (Rockville, MD). Using 96-well plates with 2,000 cells per well, HSCs were incubated in 10% FBS-supplemented DMEM for 24 hours, followed by starvation in serum-free media. After 24 hours of starvation, brivanib was added at different doses. Two hours later, 5 ng/ml PDGF-BB was added. The cells were incubated for an additional 72 hours and cell viability was measured. Each experiment was performed in three replicates at least four times.

### Statistical analysis

All animal data represent at least five (maximum of ten) independent mice and are expressed as the mean ± SEM. Differences between groups were compared using a two-tailed Student *t*-test (*P<0.05, **P<0.01). Data for LX-2 cells represent the average of three to four replicates and are expressed as mean ± SEM. Log plots for viability assay and IC_50_ calculations were obtained using GraphPad Prism software.

## Results

### Brivanib ameliorates BDL-induced liver fibrosis in mice

**Figure 1:**
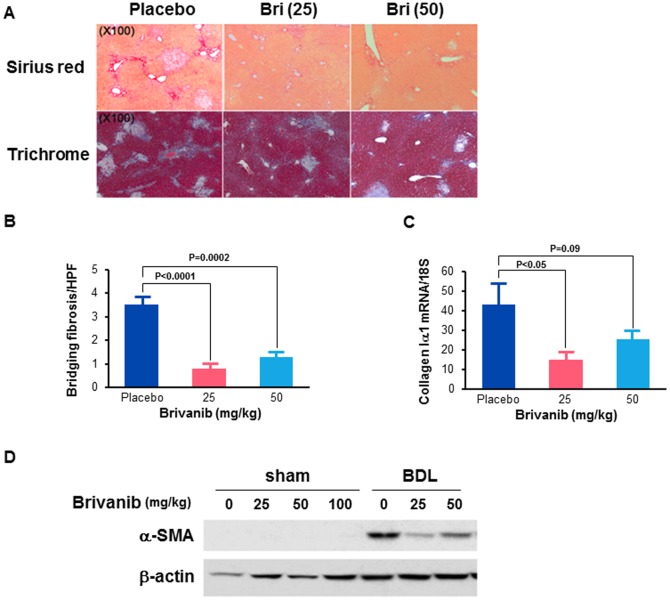
Brivanib inhibits liver fibrosis induced by bile duct ligation. (A) Histological findings at 14 days after BDL. The Sirius red and trichrome images are taken at 100×. (B) The number of bands of bridging fibrosis per high power field were counted in images obtained at 100× magnification after Sirius red staining. (C) Hepatic levels of *collagen Iα1* mRNA were measured by real time PCR in placebo, 25 mg/kg and 50 mg/kg brivanib groups at 14 days after BDL (n = 6 per group). (D) Western immunoblotting for α-SMA showing decreased whole liver α-SMA after treatment with brivanib. The expression of α-SMA in lysates extracted from liver tissue following sham ligation of the bile duct was measured by Western immunoblotting. β-actin is shown to control for loading. The sham controls confirm that brivanib does not induce liver fibrosis.

We explored the therapeutic potential of brivanib to prevent or treat liver fibrosis associated with chronic liver injury in adult male C57BL/6J mice subjected to BDL and allowed to recover for 7 days from the procedure, followed by oral administration of placebo or brivanib for another 7 days. Brivanib is excreted in large part in the bile, therefore, we initially assessed the effect of brivanib on survival after bile duct ligation. Survival after treatment with 25 mg/kg or 50 mg/kg brivanib was the same as after treatment with placebo. However, treatment with 100 mg/kg brivanib resulted in substantially shorter survival. We therefore used only the 25 mg/kg and 50 mg/kg doses for subsequent BDL experiments. Fourteen days after BDL, there was no significant difference between the placebo, 25 mg/kg brivanib or 50 mg/kg brivanib groups with regard to measures of liver injury as assessed by serum ALT levels. The ALT means and SD were: 801+/−256 IU/L; 1554+/−1423 IU/L; and 1757+/−1976 IU/L, respectively. Hepatic deposition of collagen was assessed in liver specimens by Sirius red and Masson's trichrome staining with quantitation of the numbers of bands of liver fibrosis. The number of bands of bridging fibrosis seen after staining with Sirius red was markedly reduced in the 25 mg/kg and 50 mg/kg brivanib groups respectively, compared to placebo (Fig. 1A and 1B). Trichrome staining was also reduced in both treatment groups, with a greater reduction in staining in the 50 mg/kg brivanib compared to the 25 mg/kg brivanib group (Fig. 1A). The levels of *collagen Iα1* mRNA expression were also reduced by 64% and 40% in both treatment groups compared to placebo. There was a significant reduction in *collagen Iα1* mRNA expression in the 25 mg/kg brivanib group (P<0.05) and a trend towards significance in the 50 mg/kg brivanib group (p = 0.09; one sided P value) (Fig. 1C). HSC activation, as assessed by immunoblotting for α-SMA, was reduced in both treatment groups compared to placebo; with a greater reduction in the 25 mg/kg brivanib than the 50 mg/kg brivanib group (Fig. 1D). Together, these data show that, at both the 25 mg/kg and 50 mg/kg doses, brivanib attenuates liver fibrosis and stellate cell activation induced by BDL in mice. Brivanib alone does not induce liver fibrosis; as shown in the control data from sham treated mice (Fig. 1D).

### Brivanib inhibits growth factor and growth factor receptor mRNA expression in sham control animals but shows variable effects in bile duct ligated animals

**Figure 2:**
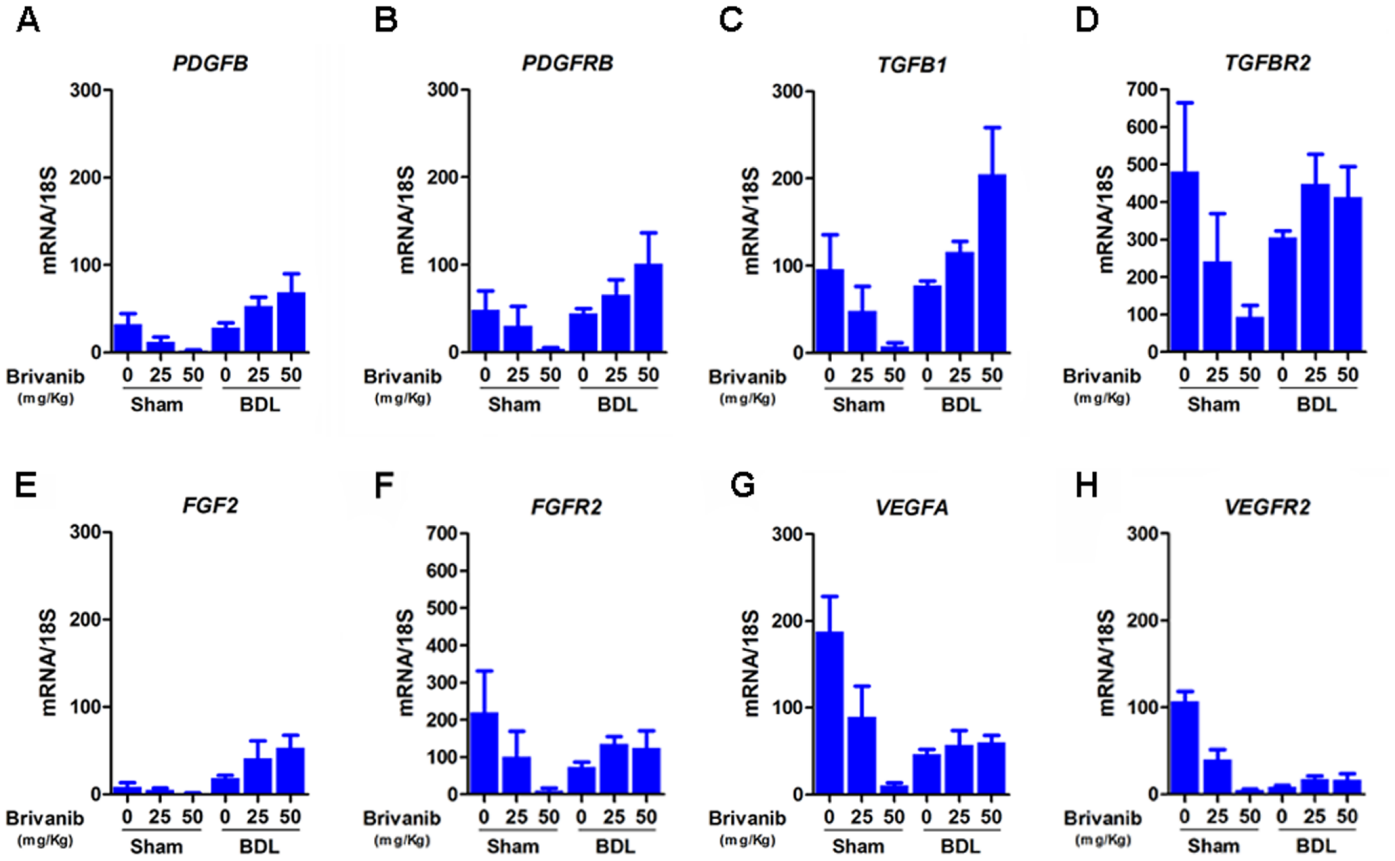
Brivanib stimulates the transcription of growth factors and their receptors in bile duct ligated mice. Hepatic levels of growth factors and growth factor receptor mRNA were measured by real time PCR in sham and BDL mice treated with no brivanib or with brivanib 25/kg or 50 mg/kg. In all of the sham experiments, higher concentrations of brivanib decreased the mRNA levels of the growth factors and their receptors. (A, B) BDL mice treated with brivanib show a dose-dependent increase in mRNA levels of *PDGFB* and *PDGFRB*. (C, D) mRNA levels of *TGFB1* increases as the concentration of brivanib increases in BDL mice; mRNA levels of *TGFBR2* are higher in BDL mice treated with 25 mg/kg and 50 mg/kg of brivanib, compared to the no brivanib group. (E, F) mRNA levels of *FGF2* and *FGFR2* are slightly higher with brivanib compared to no brivanib in BDL mice. (G, H) the mRNA levels of *VEGFA* and *VEGFR2* are not affected by brivanib treatment in BDL mice.

Figure 2 shows the results of mRNA expression as determined by real time PCR for the stellate cell activating ligands *PDGFB* and *TGFB1* and their corresponding receptors *PDGFRB* and *TGFBR2*, as well as for the major growth factors and receptors targeted by brivanib, *FGF2/FGFR2* and *VEGFA/VEGFR2*. To determine the effects of brivanib in the context of fibrosis induced by bile duct ligation, we examined expression of each growth factor and its cognate receptor in sham control mice and bile duct-ligated mice treated with 0, 25, and 50 mg/kg of brivanib. The results showed that in sham control mice, brivanib universally decreased the expression of growth factor and growth factor receptor mRNAs. The results after bile duct ligation were more variable. Bile duct ligation alone decreased expression of all the growth factors and their receptors except for *FGF2*, which was unchanged. However, treatment with brivanib increased the mRNA levels of *PDGFB*, *PDGFRB*, *TGFB1*, *TGFBR2*, *FGF2*, *FGFR2* and *VEGFR2*; in contrast there was no change in *VEGFA* in BDL mice after brivanib treatment. Overall, bile duct ligation appears to abrogate the brivanib-induced decrease in growth factor and growth factor receptor mRNA expression.

### Brivanib reduces CCl_4_-induced liver fibrosis in mice

**Figure 3:**
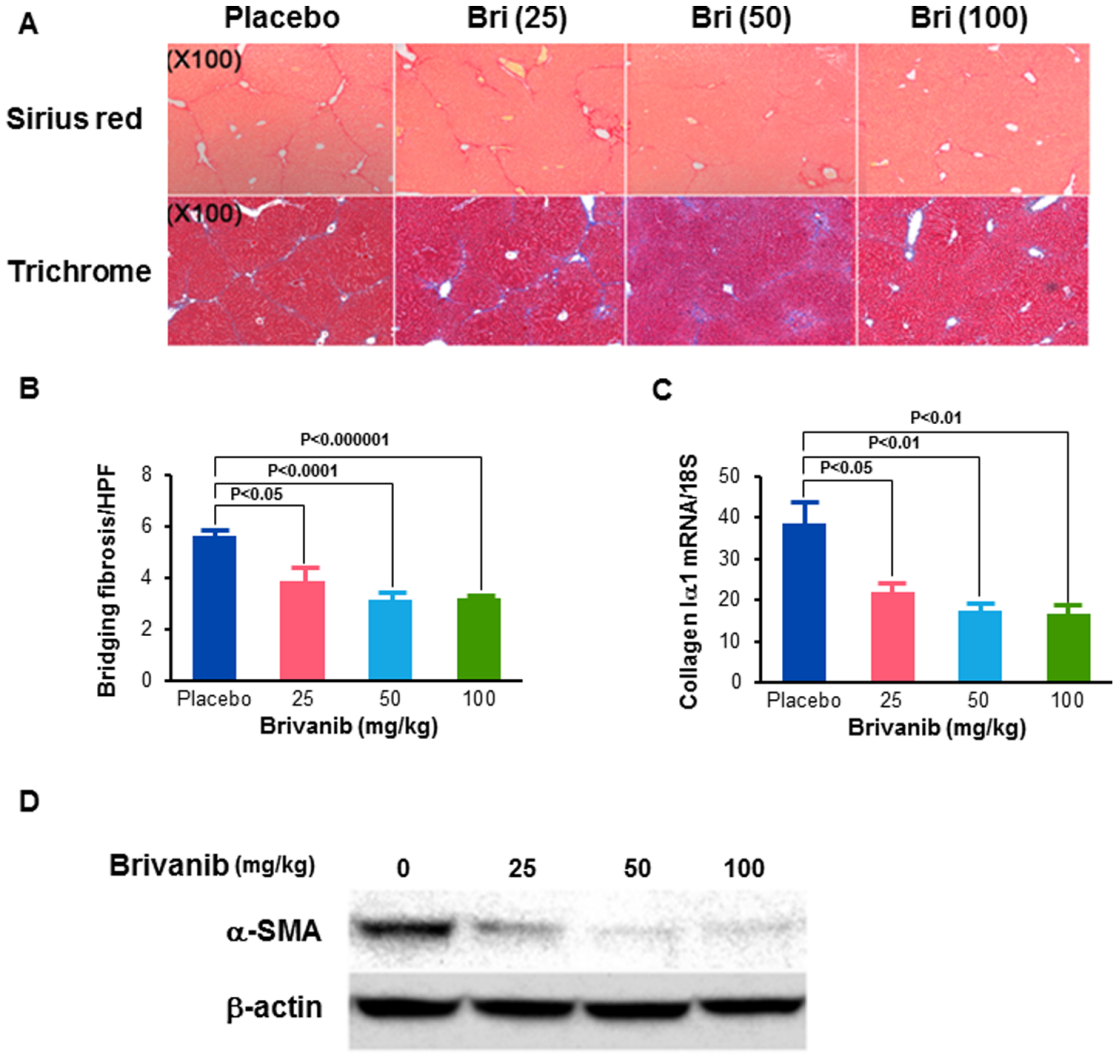
Brivanib inhibits liver fibrosis induced by carbon tetrachloride. (A) Histological analysis of livers from placebo and brivanib (25, 50, and 100 mg/kg) groups at 4 weeks after the initiation of carbon tetrachloride (CCl_4_). Pictures of Sirius red and Masson's trichrome are taken at 100× magnification. (B) The number of bands of bridging fibrosis per high power field were counted in images obtained at 100× magnification after Sirius red staining. (C) Hepatic levels of *collagen 1α1* mRNA were measured by real time PCR in placebo, 25 mg/kg, 50 mg/kg, and 100 mg/kg brivanib groups (n = 6 per group) at 4 weeks after the initiation of CCl_4_ administration. (D) Western immunoblotting for α-SMA showing decreased whole liver α-SMA after treatment with brivanib.

**Figure 4:**
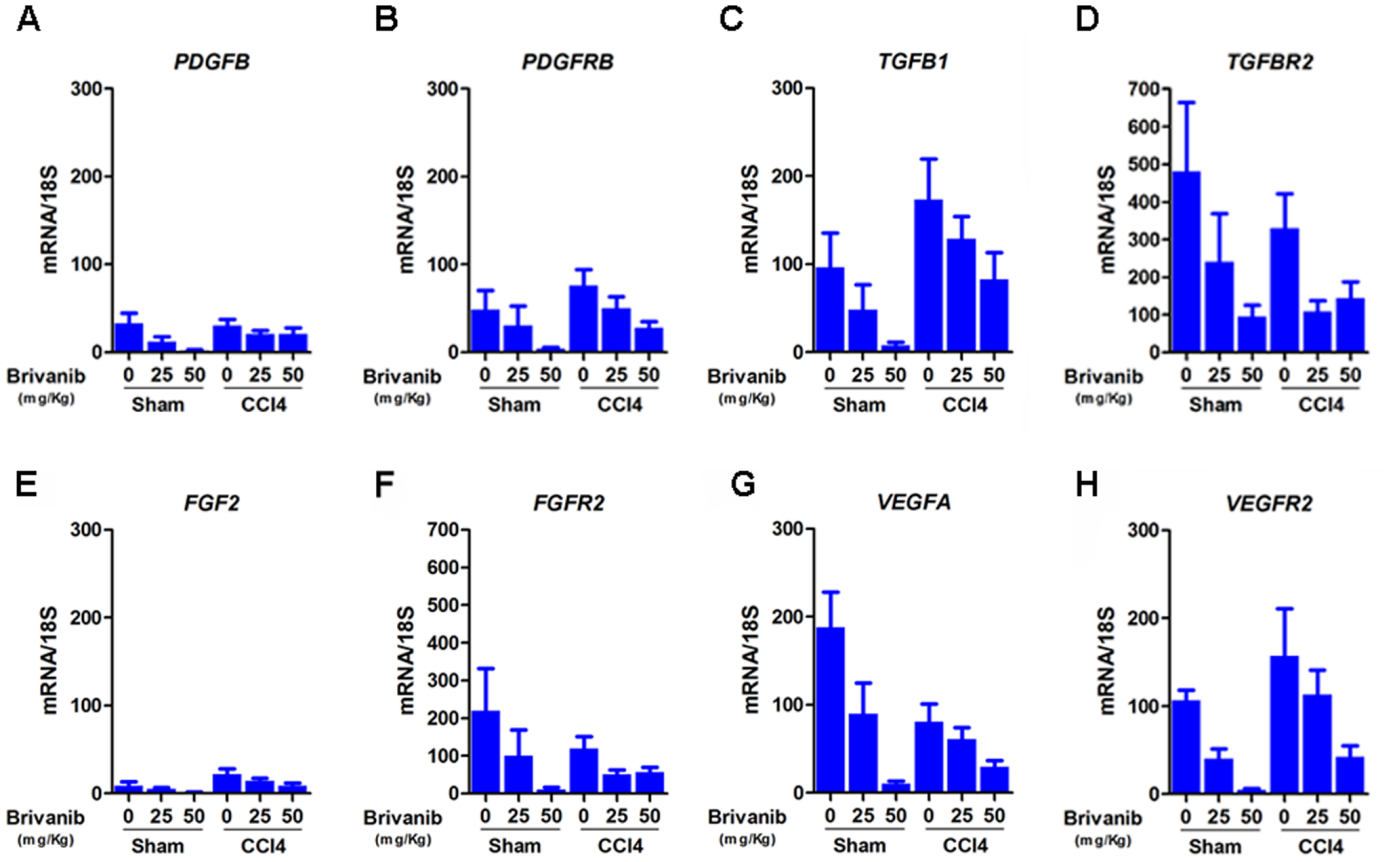
Brivanib decreases the transcription of growth factors and their receptors in carbon tetrachloride. Hepatic levels of growth factors and growth factor receptor mRNA were measured by real time PCR in sham and CCl_4_ mice treated with no brivanib or with brivanib 25 mg/kg, 50 mg/kg, or 100 mg/kg. In all of the sham experiments, higher concentrations of brivanib decreased the mRNA levels of the growth factors and their receptors. (A) mRNA levels of *PDGFB* are not affected by brivanib treatment in CCl_4_. (B) mRNA levels of *PDGFRB* decrease as the concentration of brivanib increases in CCl_4_. (C) mRNA levels of *TGFB1* decrease as the concentration of brivanib increases in CCl_4_. (D) mRNA levels of *TGFBR2* are lower in brivanib-treated CCl_4_ mice compared to no brivanib. (E) mRNA levels of *FGF2* are not affected by brivanib treatment in CCl_4_. (F) mRNA levels of *FGFR2* are lower in brivanib-treated CCl_4_ mice compared to no brivanib. (G, H) mRNA levels of *VEGFA* and *VEGFR2* decrease as the concentration of brivanib increases in CCl_4_ mice.

For our next model, we treated mice with intraperitoneal injections of CCl_4_ for 4 weeks. Since this model does not result in biliary obstruction, we were able to administer 25, 50, and 100 mg/kg doses of brivanib. The morphological changes of liver injury and fibrosis caused by CCl_4_ were visualized in liver sections stained with Sirius red and Masson's trichrome. The number of bands of bridging fibrosis per high power field counted on the Sirius red stained slides showed a progressive and significant decrease in all three brivanib groups compared with the placebo group, with a 29% decrease in the 25 mg/kg group (P<0.05), a 45% decrease in the 50 mg/kg group (*P*<0.0001) and a 43% decrease in the 100 mg/kg group (*P*<0.000001) (Fig. 3A and 3B). With trichrome staining, there were mature thin dark blue collagen bands formed in the livers of placebo control animals, while the 25 and 50 mg/kg brivanib doses resulted in lighter blue, more feathery immature collagen staining. The 100 mg/kg treated mice showed almost undetectable collagen staining (Fig. 3A). The levels of *collagen Iα1* mRNA decreased markedly with increasing brivanib dose in the 25, 50 and 100 mg/kg groups after CCl_4_ administration (Fig. 3C, P<0.05). Finally, the expression of α-SMA in liver tissue was also reduced by brivanib as assessed by Western immunoblotting, and the extent of reduction increased with increasing brivanib dose (25 mg/kg, 50 mg/kg, and 100 mg/kg) (Fig. 3D). These findings demonstrate a dose-response effect of brivanib on CCl_4_-induced liver fibrosis. Brivanib decreases the transcription of growth factors and their receptors in CCl_4_-induced liver fibrosis, as seen in the results of mRNA expression determined by real time PCR (Figure 4).

### Brivanib reduces TAA-induced liver fibrosis in mice

**Figure 5:**
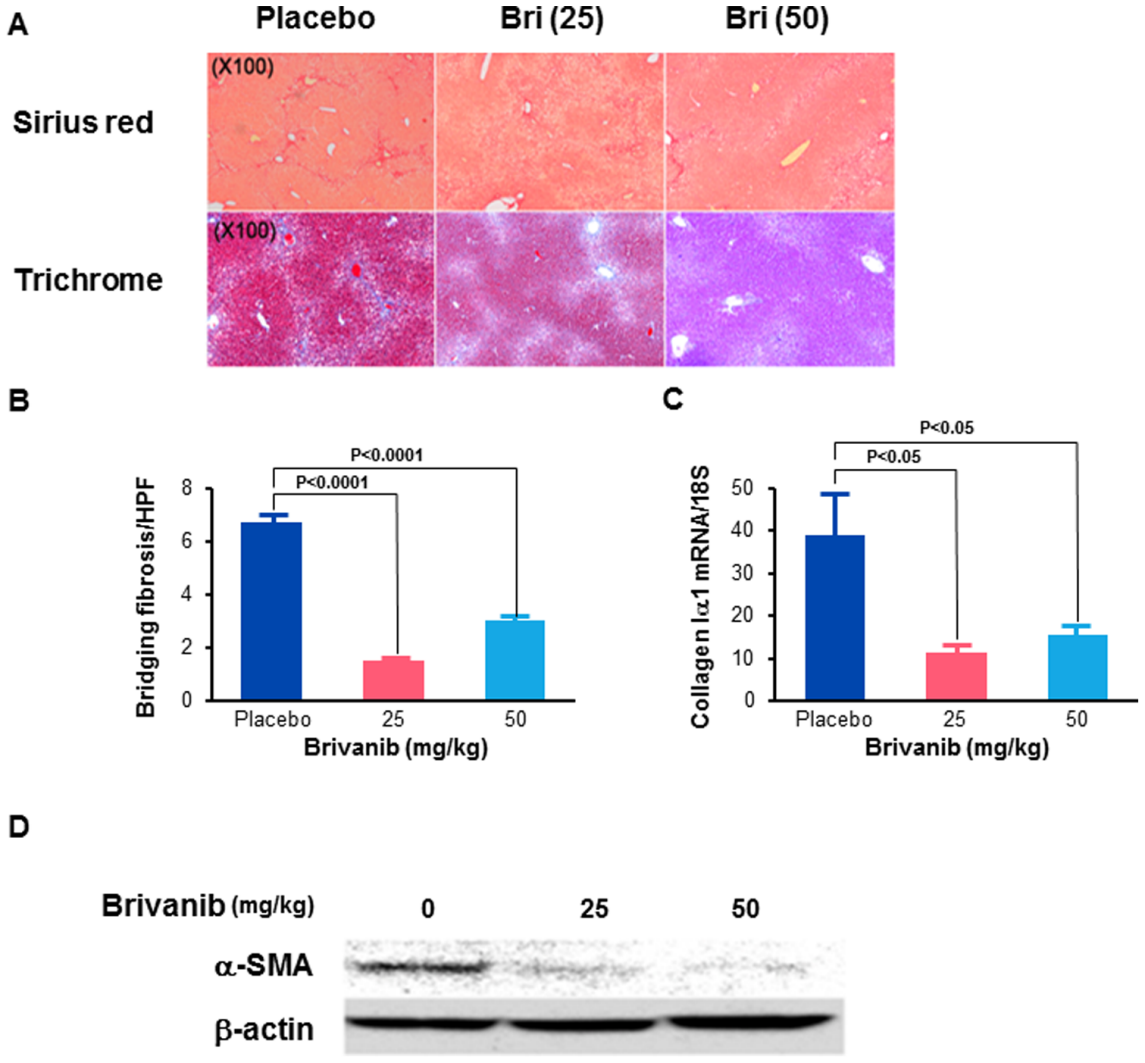
Brivanib inhibits liver fibrosis induced by thioacetamide. (A) Histological analysis of livers from placebo and brivanib-treated (25 and 50 mg/kg) groups at 4 weeks after initiation of thioacetamide (TAA). Pictures of Sirius red and Masson's trichrome staining are taken at 100×, magnification. (B) The number of bands of bridging fibrosis per high power field were counted in images obtained at 100× magnification after Sirius red staining. There was a significant decrease in the number of bands in both the 25 mg/kg and 50 mg/kg brivanib groups, compared to placebo. (C) The hepatic level of *collagen Iα1* mRNA was measured by real time PCR in placebo, 25 mg/kg and 50 mg/kg brivanib groups (n = 6 per group) at 4 weeks after the initiation of TAA. There was a substantial reduction in *collagen Iα1* mRNA at both brivanib dose levels. (D) Western immunoblotting for α-SMA showing decreased whole liver α-SMA after treatment with brivanib.

**Figure 6:**
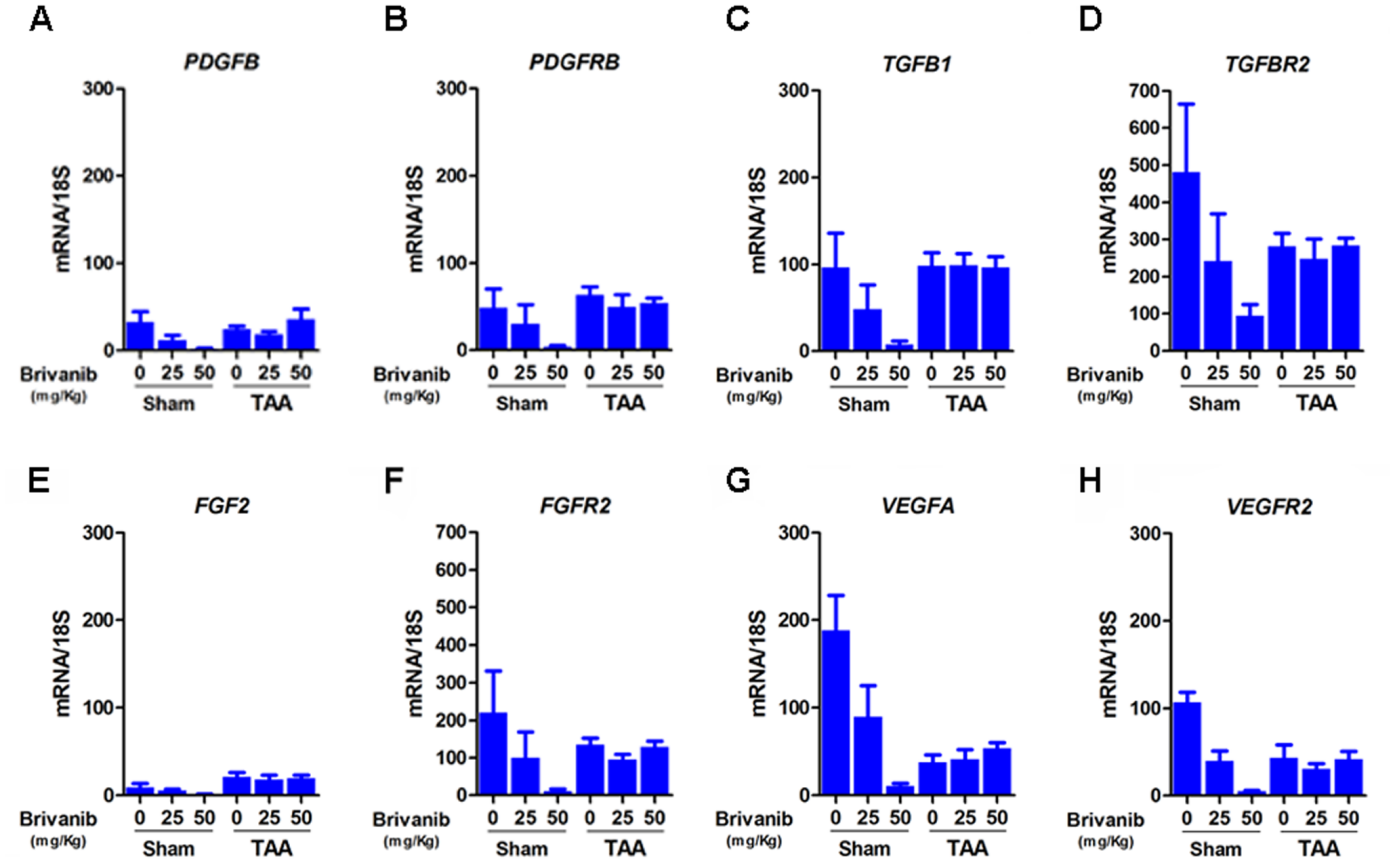
Brivanib does not affect the transcription of growth factors and their receptors in thioacetamide treated mice. Hepatic levels of growth factors and growth factor receptor mRNA were measured by real time PCR in sham and TAA mice treated with no brivanib or with brivanib 25/kg, 50 mg/kg, or 100 mg/kg. In all of the sham experiments, higher concentrations of brivanib decreased the mRNA levels of the growth factors and their receptors. mRNA levels of *PDGF*, *PDGFBR*, *TGFB1*, *TGFBR2*, *FGF2*, *FGFR2*, *VEGFA*, and *VEGFR2* are not affected by brivanib treatment of TAA mice.

Thioacetamide induces micronodular cirrhosis of the liver and has been used as a model of both prevention and treatment of liver fibrosis and cirrhosis [22]. Liver fibrosis developed during the 4 weeks of TAA treatment, and was assessed using Sirius red and trichrome staining (Fig. 5A). TAA-treated mice showed reduced Sirius red staining in both the 25 mg/kg and 50 mg/kg brivanib groups compared to placebo (Fig. 5A). Both the 25 mg/kg and 50 mg/kg brivanib groups showed reduced trichrome staining compared to the placebo group (Fig. 5A). The number of bands of bridging fibrosis was significantly reduced in both the 25 mg/kg brivanib and 50 mg/kg brivanib groups compared to the placebo group (Fig. 5B), and the expression of *collagen Iα1* mRNA was also significantly decreased in the brivanib-treated groups (Fig. 5C). Finally, expression of α-SMA as assessed by Western immunoblotting was substantially reduced in both the 25 and 50 mg/kg brivanib groups (Fig. 5D). As shown by results of real time PCR for mRNA expression, brivanib does not affect the transcription of growth factors and their receptors in thioacetamide treated mice (Figure 6).

### The PDGF and FGF2 growth factors enhance the proliferation of LX-2 human HSCs

**Figure 7:**
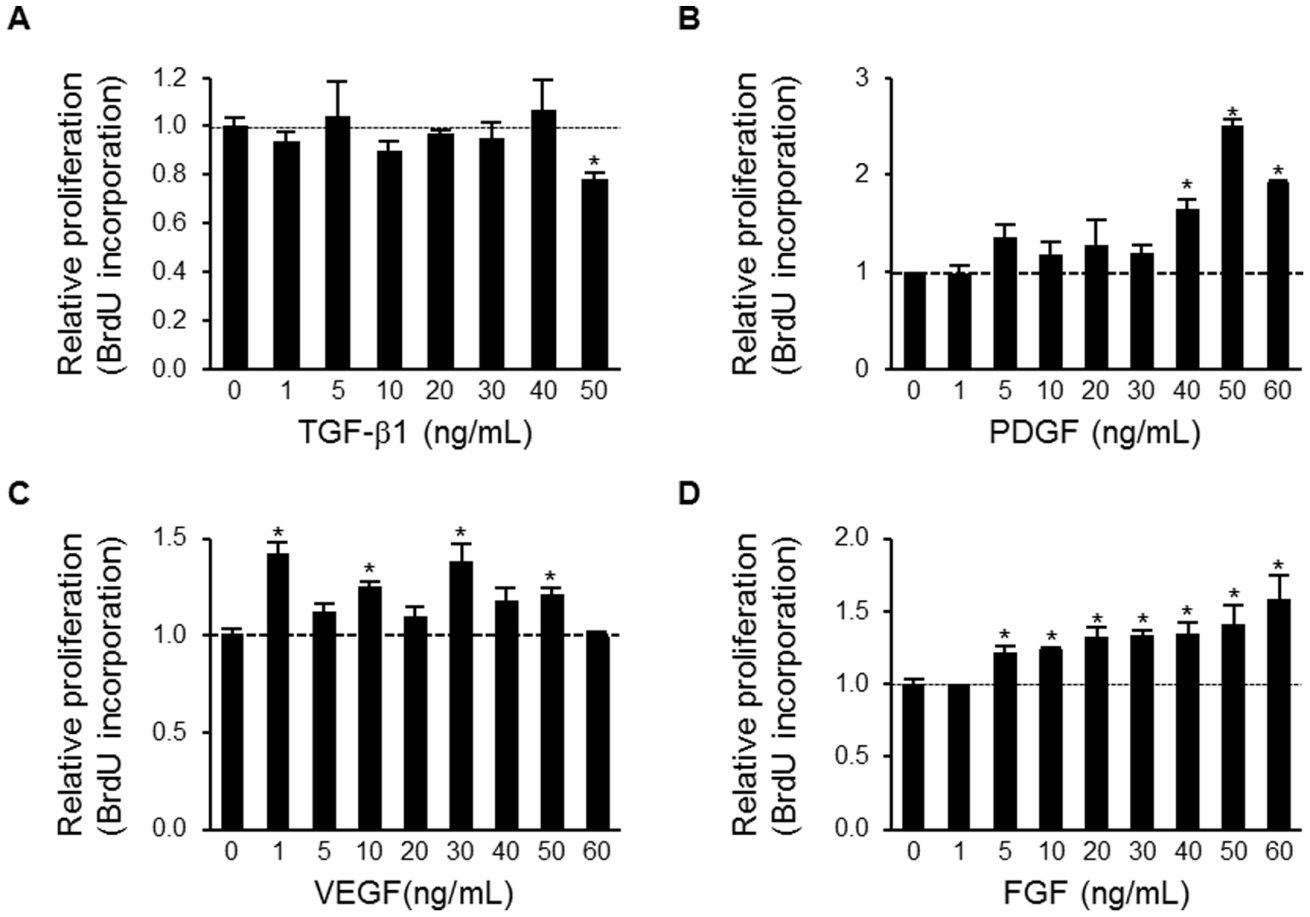
PDGF, VEGF and FGF2 all induce proliferation of human LX-2 hepatic stellate cells. Relative proliferation of LX-2 cells as assessed by BrdU incorporation after addition of TGF-β1 (A); PDGF (B); VEGF (C); or FGF (D). The LX-2 HSCs were starved for 24 hours and then treated with the respective cytokine or growth factor. BrdU incorporation was measured 72 hours later. Data shown are representative of four samples per group and are presented as mean ± SEM. *, P<0.05 (normalized to BrdU incorporation in the absence of the growth factor).

Both PDGF and FGF2 (basic FGF) have previously been shown to induce proliferation and migration of hepatic stellate cells [23]. To develop a model for testing the effect of brivanib on mechanisms of liver fibrosis, we first determined the effect of fibrogenic growth factors on proliferation of HSCs by measuring BrdU incorporation after treatment of LX-2 cells with the cytokine TGF-β1 or the receptor tyrosine kinase growth and angiogenesis factor ligands PDGF, VEGF, and FGF2. As expected, there was no effect of TGF-β1 on proliferation of LX-2 HSCs (personal communication, Dr. Scott Friedman), and actually caused a significant decrease in proliferation at the highest dose of 50 ng/mL (Fig. 7A).

On the other hand, both PDGF and FGF induced dose-dependent increases in LX-2 cell proliferation (Fig. 7B, 7D). Overall, VEGF increased proliferation, with the highest effect of a significant 41.3% increase at a dose of 1 ng/mL (P<0.008), but there was no consistent dose-response effect (Fig. 7C).

### Brivanib inhibits PDGF, VEGF, and FGF-induced proliferation of LX-2 human hepatic stellate cells

**Figure 8:**
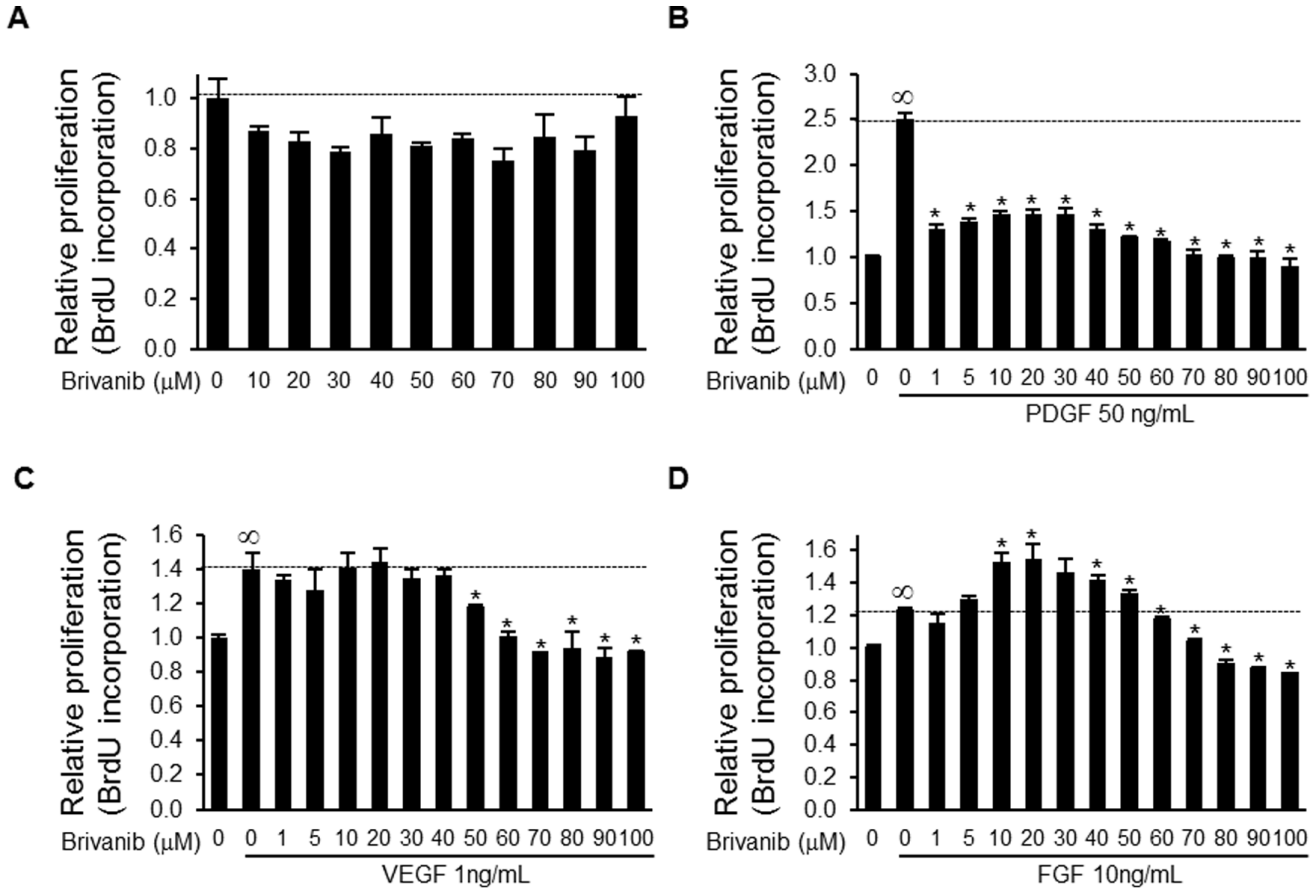
Brivanib inhibits unstimulated and PDGF, VEGF, or FGF2-stimulated LX-2 cell proliferation. The effect of brivanib on cell proliferation of LX-2 HSCs without growth factor (A). The effect of brivanib on LX-2 cell proliferation induced by 50 ng/ml PDGF (B); 1 ng/ml VEGF (C); or 10 ng/ml FGF (D). LX-2 HSCs were starved for 24 hours, brivanib was added at the indicated concentrations, and 2 hours later, the respective growth factor was added. BrdU incorporation was measured at 72 hours after the administration of growth factor. Data shown are representative of four samples per treatment group and are presented as mean ± SEM. ∞,P<0.05 (vs. without growth factor and without brivanib) and *, P<0.05 (vs. with growth factor and without brivanib).

To determine whether brivanib inhibits growth factor-induced proliferation of HSCs, we examined the effect of increasing concentrations of brivanib on LX-2 HSC proliferation under control conditions or after stimulation with concentrations of PDGF, VEGF, and FGF2 shown to induce LX-2 proliferation. Brivanib inhibited proliferation of LX-2 cells cultured in 10% serum by a maximum of about 25% at a dose of 70 µM (Fig. 8A). Somewhat unexpectedly, given the relative selectivity of brivanib for VEGF and FGF receptors, PDGF-induced LX-2 cell proliferation was substantially and significantly inhibited by brivanib even at the relatively low brivanib concentration of 1 µM (Fig. 8B). Both VEGF and FGF2-stimulated LX-2 cell proliferation were also substantially inhibited by brivanib (Fig. 8C and 8D). For both VEGF and FGF2, relatively high concentrations of brivanib appeared necessary to achieve half-maximal inhibition of LX-2 cell proliferation, approximately 50 µM in both cases. Somewhat surprisingly, brivanib doses ≤20 µM paradoxically enhanced FGF-induced LX-2 cell proliferation, whereas higher brivanib doses (≥30 µM) inhibited LX-2 cell proliferation (Fig. 8D).

### Brivanib decreases viability of PDGF-BB treated LX-2 cells

**Figure 9:**
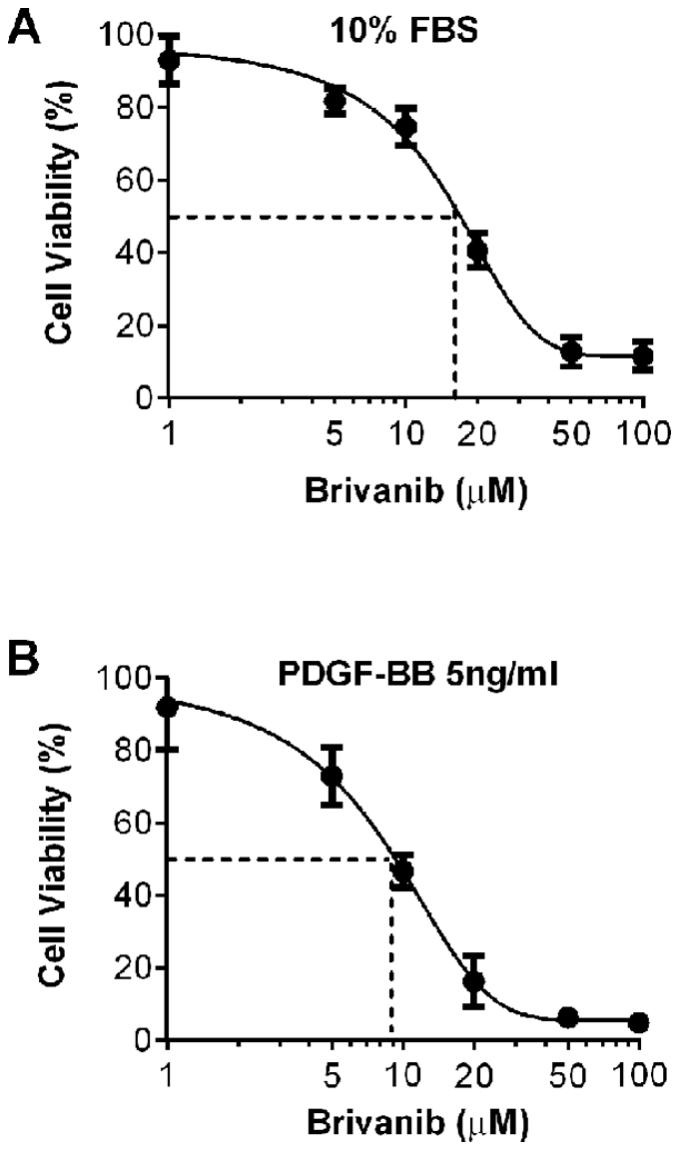
Brivanib decreases viability of PDGF-BB treated LX-2 cells. (A) The effect of increasing concentrations of brivanib on the viability of LX-2 HSCs cultured in DMEM/10% FBS was assessed using Cell Counting Kit-8 (CCK-8). Cells showed a brivanib dose-dependent decrease in viability, with half maximal inhibitory concentration (IC_50_) of 16.98 µM (95% CI 13.95 µM–20.67 µM). (B) Cells stimulated with 5 ng/ml PDGF-BB in serum-free medium showed a similar trend albeit more pronounced decrease in viability compared to cells cultured in 10% FBS. The IC_50_ of LX-2 cells cultured in serum-free media supplemented with PDGF-BB was 8.79 µM (95% CI 7.80 µM–9.99 µM).

To determine whether brivanib affects the viability profile of LX-2 hepatic stellate cells, we examined the effect of increasing concentrations of brivanib on LX-2 cell viability under control conditions of 10% FBS or after stimulation with PDGF-BB in serum-free media. LX-2 cells cultured in 10% FBS and varying concentrations of brivanib showed a dose-dependent decrease in viability (Fig. 9A). The half maximal inhibitory concentration (IC_50_) for LX-2 HSCs in DMEM/10% FBS was 16.98 µM (95% CI 13.95 µM–20.67 µM). A similar trend was seen in brivanib-treated cells cultured in serum-free medium plus 5 ng/ml of PDGF-BB, but with a more pronounced decrease in viability compared to cells cultured in 10% FBS (Fig. 9B). The IC_50_ in serum-free medium supplemented with PDGF-BB was 8.79 µM (95% CI 7.80 µM–9.99 µM).

### The inhibitory effect of brivanib on liver fibrosis is not through inhibition of TGF-β1-induced stellate cell activation

**Figure 10:**
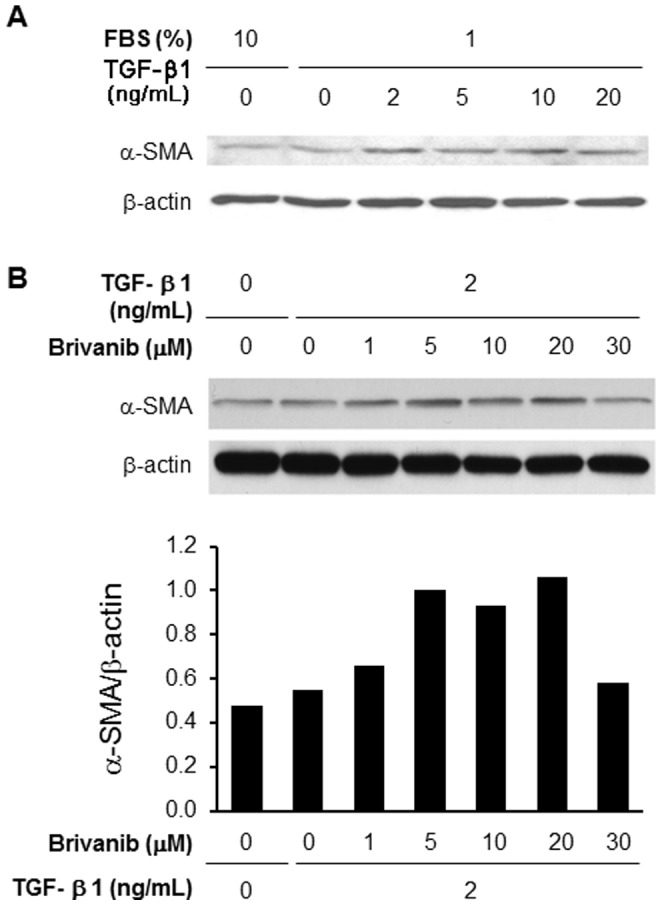
Brivanib does not inhibit TGF-β1-induced α-SMA expression in human LX-2 hepatic stellate cells. (A) The expression of α-SMA in LX-2 HSCs after TGF-β1 treatment was assessed by Western immunoblotting. HSCs were incubated with 10% or 1% FBS for 24 hours, and TGF-β1 was added at different concentrations. The lysate was extracted 24 hours after addition of TGF-β1. Peak α-SMA expression was seen after treatment with 2 ng/ml of TGF-β1. (B) To determine the effect of brivanib on TGF-β1-induced activation of HSCs as assessed by α-SMA expression, LX-2 HSCs were partially serum-starved by incubation with 1% FBS for 24 hours. Brivanib was then added at increasing concentrations 2 hours before addition of 2 ng/ml TGF-β1. Cell lysates were prepared 24 hours after adding TGF-β1 and analyzed by Western immunoblotting.

We first confirmed the known activation of LX-2 HSCs by TGF-β1 by measuring the expression of α-SMA in HSCs after TGF-β1 treatment. Doses of 2 ng/ml or greater of TGF-β1 clearly activated the expression of α-SMA (Fig. 10A). To determine the effect of brivanib on the activation of α-SMA by TGF-β1, LX-2 cells were incubated with increasing concentrations of brivanib for 1 hour before stimulation by 2 ng/ml TGF-β1. LX-2 cells showed a biphasic response to brivanib; concentrations of 5–20 µM stimulated TGF-β1-induced expression of α-SMA, while a concentration of 30 µM showed no substantial change in α-SMA level (Fig. 10B).

### The inhibitory effect of brivanib on liver fibrosis is possibly through inhibition of PDGF-BB-induced stellate cell activation

**Figure 11:**
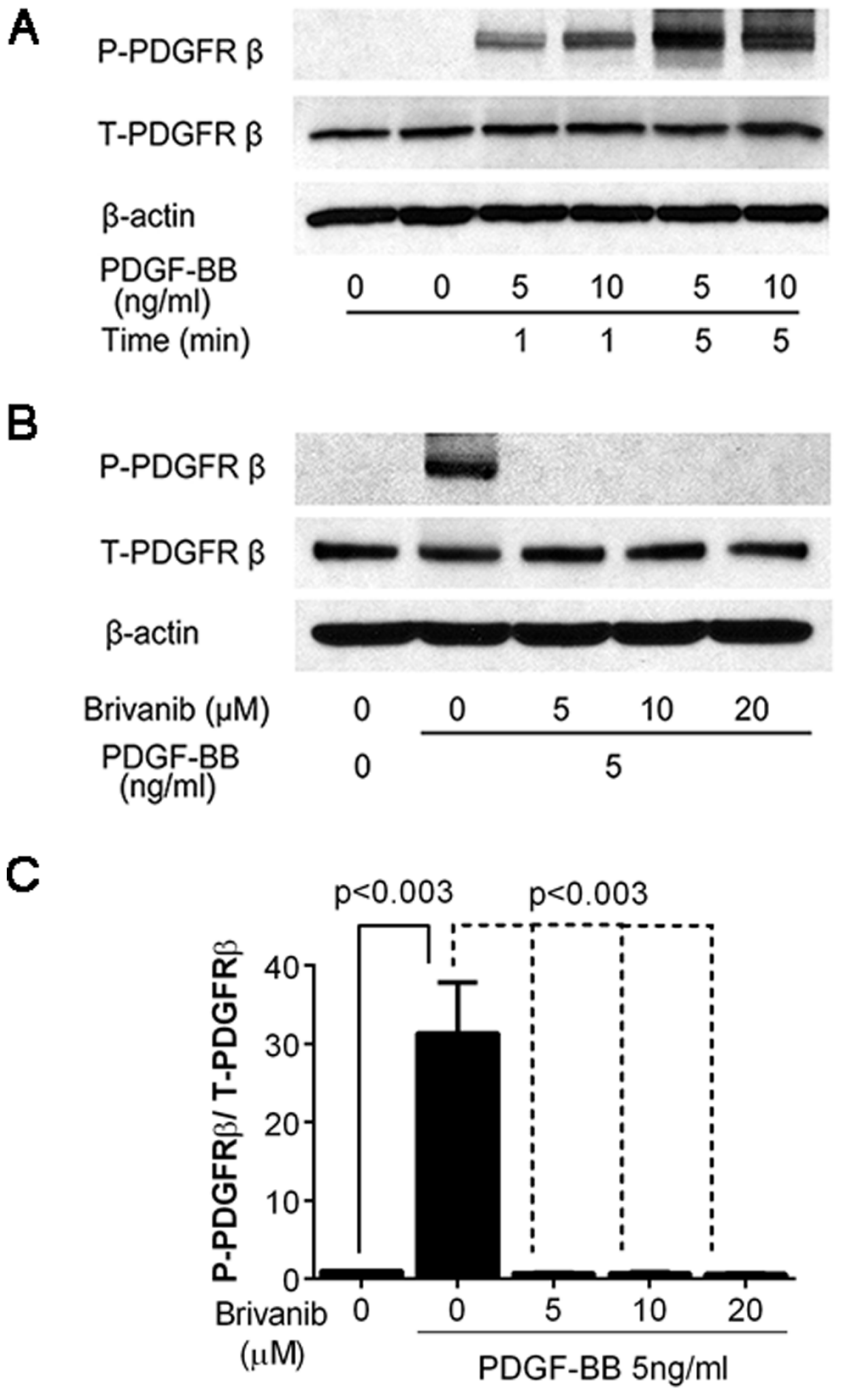
Brivanib inhibits PDGF-BB induced phosphorylation of PDGFRβ in human LX-2 hepatic stellate cells. (A) The expression of phosphorylated (P-PDGFRβ) and total PDGFRβ (T-PDGFRβ) in HSCs after PDGF-BB treatment was assessed by Western immunoblotting. HSCs were incubated in 10% FBS-supplemented DMEM for 24 hours, followed by starvation in serum-free medium for 24 hours. PDGF-BB was added at 5 or 10 ng/ml, followed by extraction of protein lysate 1 or 5 minutes after induction with PDGF-BB. Both time- and dose-dependent increase in phosphorylated PDGFRβ were seen, with minimal change in total PDGFRβ. β-actin was used as a loading control. (B) To determine the effect of brivanib on PDGF-BB induced phosphorylation of PDGFRβ, LX-2 cells were serum starved, followed by treatment with 5, 10 or 20 µM of brivanib. Two hours after brivanib treatment, 5 ng/ml PDGF-BB was added, and protein lysates prepared after 5 minutes of PDGF-BB exposure. All doses of brivanib tested inhibited PDGF-BB induced phosphorylation of PDGFRβ. (C) Ratio of phosphorylated PDGFRβ relative to total PDGFRβ. Prior to calculation of the P-PDGFRβ/T-PDGFRβ ratio, protein levels were first normalized to β-actin, and then to the P-PDGFRβ or T-PDGFRβ level in their respective control studies. Data represents the mean of four replicate studies ± SEM.

Next, we examined the effect of brivanib on PDGF-BB induced stellate cell activation. We measured the expression of phosphorylated PDGFRβ in HSCs after PDGF-BB treatment. Doses of 1 ng/ml and 5 ng/ml clearly increased the level of phosphorylated PDGFRβ as early as 1 and 5 minutes after treatment (Fig. 11A). Brivanib abrogated PDGF-BB induced phosphorylation of its cognate receptor even at the lowest tested dose of 5 µM (Fig. 11B). Brivanib does not affect the level of total PDGFRβ, but instead is involved in posttranslational modification of the receptor through phosphorylation as shown by the profound decrease in phosphorylated PDGFRβ relative to total PDGFRβ (p-value<0.003) after treatment with brivanib doses ranging from 5 to 20 µM (Fig. 11C).

## Discussion

While brivanib inhibits VEGF and FGF, both of which are related to liver fibrosis, the effect of brivanib on liver fibrosis has not been previously studied. Our results suggest that brivanib inhibits liver fibrosis through effects on multiple signaling pathways. Our findings demonstrated that: 1) brivanib reduced mouse liver fibrosis induced by bile duct ligation, carbon tetrachloride, and thioacetamide; 2) brivanib decreased cell proliferation induced by treatment of human hepatic stellate cells with PDGF, VEGF and FGF; 3) brivanib abrogated PDGF-BB mediated phosphorylation of its cognate receptor. These novel findings of inhibition of liver fibrosis and stellate cell proliferation by brivanib suggest a potential antifibrotic role for brivanib.

Our findings are consistent with the known potentiation of liver fibrosis by VEGF and FGF. We demonstrated that brivanib reduced mouse liver fibrosis induced by bile duct ligation, treatment of CCl_4_, and TAA. In a previous study, PTK787/ZK222584 (PTK/ZK), a pan-VEGFR tyrosine kinase inhibitor, attenuated collagen deposition and α-SMA expression in carbon tetrachloride-induced fibrosis in a mouse liver fibrosis model [24]. In another study, liver fibrosis resulting from chronic CCl_4_ exposure was markedly decreased in the livers of FGF1/FGF2-deficient mice [13]. These results suggest that both VEGF and FGF play critical roles in liver fibrosis.

Fibrosis in PDGF-C transgenic mice, as demonstrated by staining and hydroxyproline content, is preceded by activation and proliferation of hepatic stellate cells, as shown by collagen, α-smooth muscle actin and glial fibrillary acidic protein staining. Between 8 and 12 months of age liver adenomas and hepatocellular carcinomas develop [3]. PDGF is the most important cytokine responsible for HSC proliferation [25], [26], [27]. VEGF has also been shown to induce HSC proliferation [24]. In this study, we showed that FGF, but not TGF-β1, also increased HSC proliferation. Treatment with brivanib inhibited HSC proliferation after PDGF, VEGF and FGF treatment. TGF-β1 is a strong activator of HSC and the TGF-β1 signaling pathway plays an important role in liver fibrosis [12], [26]. Liu et al also demonstrated that PTK/ZK, a potent tyrosine kinase inhibitor that blocks the vascular endothelial growth factor receptor, significantly inhibits PDGF receptor expression, inhibits the TGF-β1-induced expression of VEGF and VEGFR1 and also down regulates TGF-β receptor expression in rat HSC, suggesting that there is substantial crosstalk between the VEGF, PDGF, and TGF-β signaling pathways in HSCs. In this study, brivanib had a biphasic effect but did not show strong inhibition of the TGF-β-induced activation of α-SMA in human HSCs.

Previous studies have characterized the effect of brivanib on the VEGF and FGF signaling pathways [14], [16], [17], [18], [19], but less is known about the effect of brivanib on PDGF signaling. Our findings that brivanib inhibits not only VEGF- and FGF-mediated proliferation of HSCs, but also inhibits PDGF-induced proliferation, together with our other findings that brivanib abrogates phosphorylation of the PDGFβ receptor, suggest the need to further characterize the modulation of PDGF signaling by brivanib.

Our findings demonstrate that brivanib reduces liver fibrosis in three different animal models, and inhibits growth factor-induced stellate cell proliferation and activation. Although brivanib has previously been demonstrated to decrease tumorigenesis in several cancers including HCC through the regulation of VEGF and FGF signaling pathways, the effects of brivanib on liver fibrosis had not been studied [14], [17], [28]. Our findings suggest that brivanib may have substantial therapeutic potential as an inhibitor of liver fibrosis.

## Conclusion

In this study we have shown that brivanib reduces liver fibrosis in three different animal models through effects on PDGF, VEGF and FGF signaling. The inhibitory effect of brivanib on liver fibrosis is not through inhibition of TGF-β1-induced stellate cell activation. Brivanib may represent a novel therapeutic approach to treatment of liver fibrosis and prevention of liver cancer.
