# Correction: Brivanib Attenuates Hepatic Fibrosis *In Vivo* and Stellate Cell Activation *In Vitro* by Inhibition of FGF, VEGF and PDGF Signaling

**DOI:** 10.1371/journal.pone.0142355

**Published:** 2015-11-03

**Authors:** Ikuo Nakamura, Kais Zakharia, Bubu A. Banini, Dalia S. Mikhail, Tae Hyo Kim, Ju Dong Yang, Catherine D. Moser, Hassan M. Shaleh, Sarah R. Thornburgh, Ian Walters, Lewis R. Roberts

The following information is missing from the Funding section: This research was supported by a National Institutes of Health (NIH) grant, 3R01CA165076-03S2, Research Supplement to Promote Diversity in Health-Related Research, to Hassan M. Shaleh.
